# The rs1024611 in the CCL2 gene and risk of gynecological cancer in Asians: a meta-analysis

**DOI:** 10.1186/s12957-018-1335-4

**Published:** 2018-02-20

**Authors:** Shuying He, Xiuzhen Zhang

**Affiliations:** 1 Department of Obstetrics and Gynecology, China XD Group Hospital, No. 97 Fengdeng Road, Lianhu District, Xi’an, China; 2 1st Department, Gynecology Oncology, Shaanxi Provincial Tumor Hospital, No. 309, Yantaxi Road, Xi’an, 710061 China

**Keywords:** Gynecological cancer, CCL2, CCL2-2518A/G, Polymorphism, Meta-analysis

## Abstract

**Background:**

The -2518A/G (rs1024611) polymorphism of the CCL2 (C-C motif chemokine ligand 2), also known as MCP-1 (monocyte chemotactic protein-1) gene, has been reported to be associated with increased gynecological cancer risk, but the results are conflicting.

**Methods:**

In this analysis, 1089 cases and 1553 controls from six publications were used to investigate the association between CCL2-2518A/G (rs1024611) polymorphism and the risk of gynecological cancer with a meta-analytic approach. Studies published on EBSCO, EMBASE, Web of Science, PubMed, SpringerLink, ScienceDirect, Weipu, and CNKI databases were identified (last update was on November 3, 2015). Six articles focused on the association between CCL2-2518A/G (rs1024611) polymorphism, and gynecological cancer risk was selected and data were extracted. The cancer type included endometrial cancer (*n* = 1), breast cancer (*n* = 2), ovarian cancer (*n* = 2), and cervical cancer (*n* = 1). All statistical analyses were performed using the STATA version 12.0 software.

**Results:**

The meta-analysis showed that CCL2-2518A/G (rs1024611) polymorphism is associated with risk of gynecological cancer (GG vs AG + AA, OR = 1.55, 95%CI = 1.07–2.24, *P* < 0.05; AA vs GG, OR = 0.59 95%CI = 0.38–0.92, *P* < 0.05). Notably, the subgroup analysis demonstrated that the genotype AA is associated with a reduced gynecological cancer risk in Asians, but an increased risk when compared to AG in Europeans.

**Conclusions:**

Our data demonstrated the CCL2-2518A/G (rs1024611) polymorphism is significantly associated with risk of gynecological cancer, and the association differs by ethnicity.

## Background

Gynecological cancer, including breast cancer (BC), endometrial carcinoma (EC), cervical cancer (CC), and ovarian cancer (OC), is the leading cause of cancer-related death in women. Currently, breast cancer is the most frequent among women with an estimated 1.67 million new cases diagnosed worldwide in 2012 [1]. Cervical cancer is the fourth most common cancer in women. According to GLOBOCAN 2012 statistics, there were approximately 266,000 women who died of cervical cancer worldwide [2].

Chemokines are proteins with low molecular weight (approximately 8–12 KD) that can induce leukocytes, including monocytes, neutrophil granulocytes, lymphocytes, tumor-associated macrophages (TAMs), natural killer (NK), and dendritic cells, into the area with infection. Chemokines and their receptors are associated with inflammation, cancer, allergy, autoimmunity, and AIDS [3]. Several studies showed that they play a critical role in pathological and physiological functions of the human body [4].

MCP-1 (monocyte chemotactic protein-1), also known as C-C motif chemokine ligand 2 (CCL2), is an inflammatory or inducible chemokine which is secreted by vascular endothelial cell (VEC) through exogenous lipopolysaccharide (LPS) and endogenous inflammatory factor (IL-1 and TNF-α) stimulation [5]. CCL2 and its receptor (CC chemokine receptor, CCR2) are pro-inflammatory mediators and chemoattractant that regulate the development and progression of tumor through migration and infiltration of monocytes or TAMs and CCL2/CCR2 axis. [6–8]. Epidemiology studies have shown the relationship between CCL2 level and atherosclerosis [9]. Animal model of CCL2 knockout mouse also showed decreased arterial lipid level and less infiltration of monocyte, which could alleviate the state of an illness or the risk of morbidity [10]. It has also been reported that the microenvironment regulated by chemokine was preferable to promote the proliferation of cancer cell and accelerate the process of cancerogenesis, which mechanisms include infiltrating into cancer tissue, promoting the proliferation of cancer cells through growth factor, inducing the neovascularization, and restraining the immune response during cancer [11].

Numerous studies that investigated the role of the -2518A/G (rs1026611) polymorphism in the MCP-1 (CCL2) gene have suggested that it is associated with cancer susceptibility, including breast cancer, cervical cancer, endometrial carcinoma, cervical cancer, and ovarian cancer, and might be a risk factor for cancer [12]. The rs1024611 is located in the promoter region of CCL2, and its function is complex. Previous study [13] reported that the rs1024611 is associated with allelic expression imbalance of CCL2, and there was higher level of CCL2 expression associated with the rs1024611G. However, the results reported so far remain inconclusive. We conducted a meta-analysis to further explore the association between CCL2 polymorphism and gynecological cancer risk.

## Methods

This systematic review was conducted and reported according to the preferred reporting items for systematic reviews and meta-analyses (PRISMA) [14].

### Study identification and selection

A systematic literature search was initiated in the EBSCO, EMBASE, Web of science, PubMed, SpringerLink, ScienceDirect, Weipu, and Chinese National Knowledge Infrastructure (CNKI) databases to identify articles that evaluated the association between polymorphism of the CCL2 gene and gynecological cancer risk (last update was on November 3, 2015). The search keywords were as follows: “cancer or tumor or carcinoma” in combination with “SNP or allele or polymorphism” and in combination with “CCL2 or chemokine (C-C motif) ligand 2 or MCP-1 or Monocyte chemoattractant protein 1.” There was a limit on the language of the article.

**Fig. 1:**
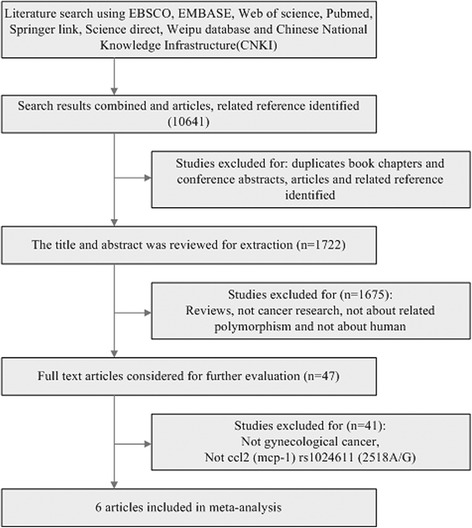
The flow chart of the article screening process

The process of the article selection was shown in Fig. 1, which included a hierarchical approach based on title, abstract, and full-text reading. Inclusion criteria of the eligible studies were as follows: case-control studies, cases were patients with gynecologic cancer (inclusive BC, EC, UCC, OC), controls consisted of healthy individuals, articles were used to evaluate the association between CCL2-2518A/G (rs1024611) polymorphism and gynecologic cancer (tumor) risk, and OR and 95% CI of the genotype or allele were reported in the studies. Reviews, duplicate book chapters, conference abstracts, and animal studies were excluded. The quality of these studies was assessed according to the Ottawa scale [15].

### Data extraction

Two investigators rigorously assessed all eligible studies based on the inclusion criteria. Date extraction was independently performed by both investigators and included the name of first author, year of publication, study country, ethnicity of the study subjects, age of controls and cases, number of controls and cases, definition of case, genotyping methods, genotype, and allele.

### Statistical analysis

Hardy-Weinberg equilibrium (HWE) in controls was examined by asymptotic Pearson’s chi-square test. The association between CCL2-2518A/G (rs1024611) polymorphism and risk of cancer was estimated with OR (odds ratio) and 95% CI in individual case-control study. Five genetic models including recessive genotype (AA vs AG + GG), dominant genotype (GG vs AG + AA), heterozygous genotype (AA vs AG), homozygote genotype (AA vs GG), and allele genotype (A vs G) were explored to estimate the effects of the MCP-1-2518A/G (rs1024611) polymorphisms on cancer risk.

The heterogeneity among studies was assessed by a *χ*^2^-based *Q* test, and the pooled OR was calculated with a fixed-effects or random-effects model according to the heterogeneity test result. The pooled OR was calculated with a fixed-effects model when the *P*_het_ > 0.05. In contrast, a random-effects model was used when the *P*_het_ ≤ 0.05. Publication bias was assessed with Begg’s funnel plots (*Pr* > |*z*|) and Egger’s test (*Pr* > |*t*|). Subgroup analysis by ethnic group was also performed to evaluate the ethnicity-specific effect. All statistical analyses were performed using the STATA version 12.0 software.

## Results

### Study inclusion and characteristics

The search and selection criteria were showed in Fig. 1. A total of 10,641 articles were identified through an initial search of databases including EBSCO, EMBASE, Web of Science, PubMed, SpringerLink, ScienceDirect, Weipu, and CNKI. Once the duplicate articles, book chapters, and conference abstracts were excluded, 1722 articles remained. Then, we excluded those articles that were not cancer research, not related to polymorphisms in human, and 47 articles remained. Finally, 6 articles were selected into this study after 41 articles that were not related to CCL2-2518A/G (rs1024611) polymorphisms and gynecological cancer were excluded. The characteristics of the 6 studies included in this meta-analysis are shown in Table 1. There were 1 study of European population and 5 studies of Asian population, including a total of 1089 cases and 1553 controls. The cancer type included endometrial cancer (*n* = 1) [16], breast cancer (*n* = 2) [12, 17], ovarian cancer (*n* = 2) [18, 19], and cervical cancer (*n* = 1) [20]. In one study [16], the frequency of genotype in the control deviated from HWE (*χ*^2^ = 4.12, *P* < 0.05). Genotype and allele distribution of CCL2-2518A/G (rs1024611) polymorphisms in gynecologic cancer patients and control are showed in Table 2. The quality scores of these studies were between 5 and 7.

**Table 1:** Characteristics of the studies included in the analysis

Authors	Year of publication	Country	Host ethnicity	Age, years mean ± SD		Sample *n*		Genotyping
				Case	Controls	Case	Controls	
Rukset A.et al	2010	Turkish	Asian	57.75 ± 4.98	55.23 ± 6.55	50	211	PCR-RFLP
Vasudha S.et al	2015	India	Asian	49.05 ± 11.70	49.03 ± 11.69	200	200	PCR
Łukasz K.et al	2011	Poland	European	60.9 ± 10.1	59.7 ± 11.2	160	323	PCR
Xin W.et al	2014	China	Asian	54.6 ± 12.2	53.2 ± 11.7	257	273	PCR-RFLP
Wu H.et al	2010	China	Asian	54.2 ± 12.1		147	253	PCR-RFLP
Li L.et al	2015	China	Asian	55.6 ± 12.8	54.9 ± 13.7	275	293	PCR-RFLP

*SD* standard deviation, *PCR-RFLP* polymerase chain reaction-restriction fragment length polymorphism

**Table 2:** Genotype and allele distribution of ccl2 polymorphisms in gynecologic cancer patients and controls

SNP	Study	Case					Control					HWE	
		AA	AG	GG	A	G	AA	AG	GG	A	G	*X* ^2^	*p*
rs1024611	RA et al.	26	17	7	69	31	124	82	5	330	92	4.12	0.04
	VS et al.	86	83	31	255	145	97	86	17	280	120	0.11	0.74
	TK et al.	89	54	17	232	88	154	145	24	453	193	1.65	0.2
	Xin et al.	30	115	112	175	339	47	135	91	229	317	0.06	0.8
	Wu et al.	23	81	43	127	167	33	132	88	198	308	2.29	0.13
	Li et al.	37	120	118	194	356	58	140	95	256	330	0.24	0.62

*SNP* single nucleotide polymorphism, *HWE* Hardy-Weinberg equilibrium

### Meta-analysis

#### Quantitative data synthesis and hypothesis testing

**Fig. 2:**
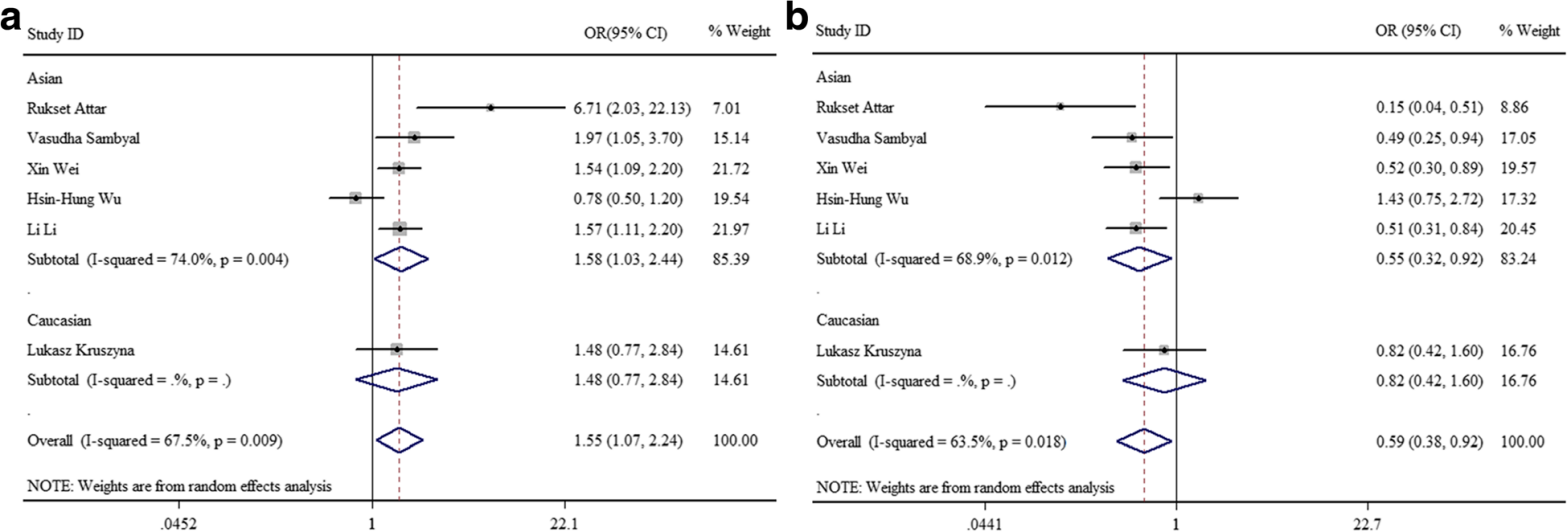
The forest plots of the association between CCL2-2518A/G polymorphism and gynecological cancer risk. **a** CCL2-2518A/G dominant (GG vs AG + AA) model. **b** Heterozygous (AA vs GG) model. CCL2 C-C motif chemokine ligand 2, ORs odds ratios

A meta-analysis of five comparative genetic models of -2518A/G polymorphisms and gynecological cancer risk was performed. To define the appropriate model to be used, we interrogated the heterogeneity of the five genetic models. The heterogeneity result of AA vs AG + GG, GG vs AG + AA, AA vs GG, and A vs G were significant, with *P*_het_ value < 0.05 (*I*^2^ = 53.90%), 0.01 (*I*^2^ = 67.50%), 0.02 (*I*^2^ = 63.50%), and 0.01 (*I*^2^ = 66.00%) respectively, and the random-effects model was used to synthesize the data. AA vs AG model used the fixed-effects model. Indeed, ORs of dominant GG vs AG + AA model and heterozygous AA vs GG model were 1.55 (95%CI = 1.07–2.24, *P* < 0.05) and 0.59 (95%CI = 0.38–0.92, *P* < 0.05) respectively, indicating a significant association between CCL2-2518A/G and gynecological cancer risk (Fig. 2a, b; Table 3). On the contrary, no association between CCL2-2518A/G and gynecological cancer risk was noted in the AA vs AG + GG (OR = 0.87, 95%CI = 0.65–1.16, *P* > 0.05), AA vs AG (OR = 1.01, 95%CI = 0.83–1.23, *P* > 0.05), and A vs G (OR = 0.83, 95%CI = 0.68–1.02, *P* > 0.05) models (Table 3).

**Table 3:** Meta-analysis of five comparative genetic models of -2518A/G polymorphisms on gynecological cancer risk

Genetic model	OR (95% CI)	*Z*	*P* value	*I*^2^%	*P*_het_	Effect model	Begg’s test *p* > │*z*│	Egger’s test *p* > │*t*│
AA vs AG + GG	0.87 (0.65, 1.16)	0.96	0.34	53.90	0.05	Random	0.85	0.64
GG vs AG + AA	1.55 (1.07, 2.24)	2.33	0.03	67.50	0.01	Random	0.57	0.31
AA vs AG	1.01 (0.83, 1.23)	0.08	0.93	32.80	0.19	Fixed	0.85	0.61
AA vs GG	0.59 (0.38, 0.92)	2.34	0.02	63.50	0.02	Random	0.85	0.53
A vs G	0.83 (0.68, 1.02)	1.73	0.08	66.00	0.01	Random	0.35	0.95

*OR* odds ratio

#### Subgroup analysis

**Fig. 3:**
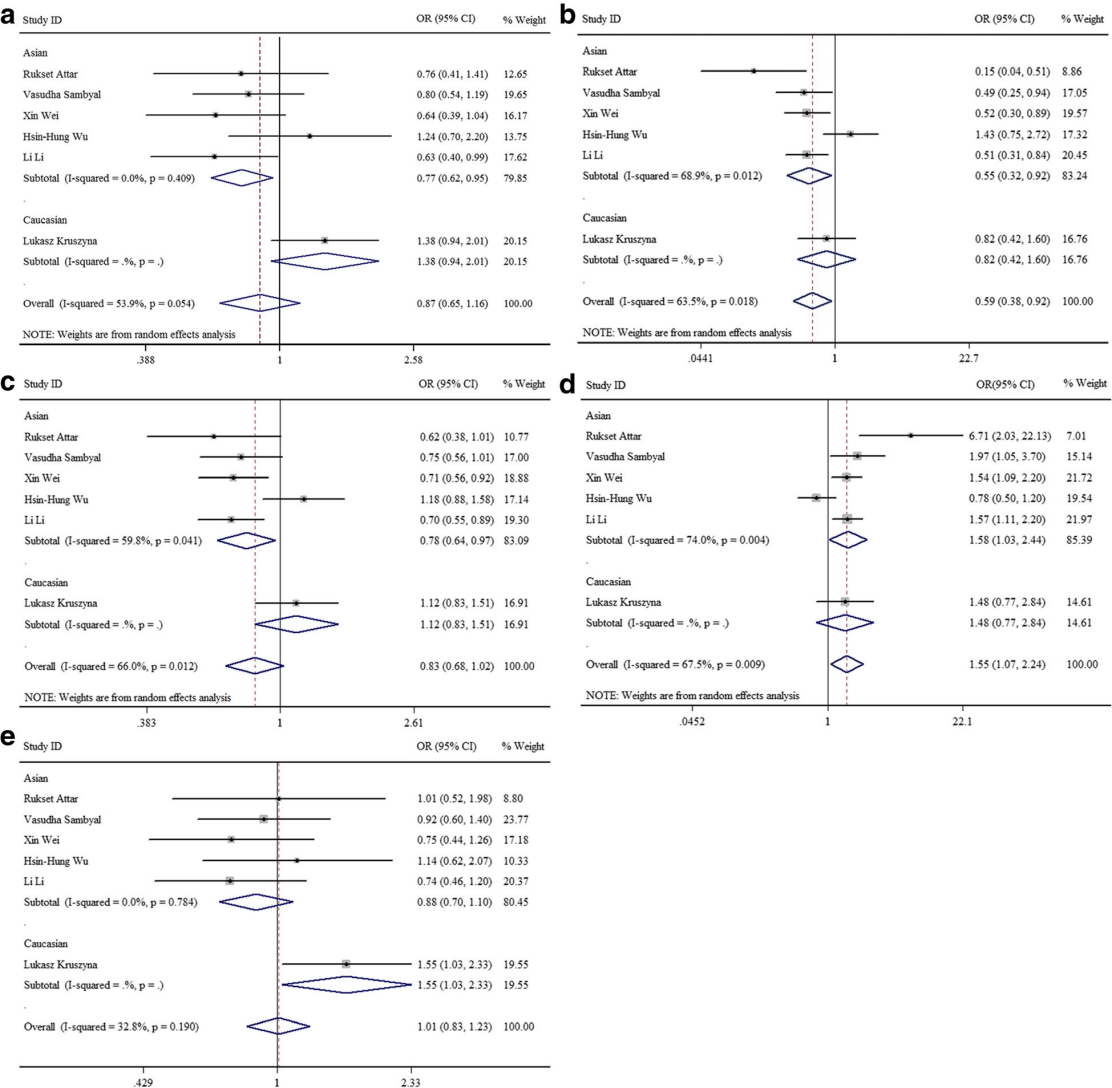
The forest plots of the association between CCL2-2518A/G polymorphism and gynecological cancer risk by ethnicity. **a** CCL2-2518A/G recessive (AA vs AG + GG) model. **b** CCL2-2518A/G homozygote (AA vs GG) model. **c** CCL2-2518A/G allele. **d** CCL2-2518A/G dominant (GG vs AG + AA) model. **e** CCL2-2518A/G heterozygous (AA vs AG) model. CCL2 C-C motif chemokine ligand 2, ORs odds ratios

According to the study population, we also investigated the association by the ethnicity in the subgroup analysis with five genetic models. The results of subgroup analysis showed that AA vs AG + GG (OR = 0.77, 95%CI = 0.62–0.95, *P* < 0.05), AA vs GG (OR = 0.55, 95%CI = 0.32–0.92, *P* < 0.05), and A vs G (OR = 0.78, 95%CI = 0.64–0.97) of CCL2-2518A/G (rs1024611) were all associated with the reduced risk of gynecological cancer in Asians (Fig. 3a–e), There was also a reduced risk of gynecological cancer in AA genotype compared with GG (OR = 0.59, 95%CI = 0.38–0.92). In contrast, genetic models of GG vs AG + AA (OR = 1.58, 95%CI = 1.03–2.44, *P* < 0.05) in Asians and AA vs AG (OR = 1.55, 95%CI = 1.03–2.33, *P* < 0.05) in Europeans both showed an increased risk of gynecological cancer, and the overall population OR of the GG vs AG + AA was 1.55 (95%CI = 1.07–2.24, *P* < 0.05) (Fig. 3d, e).

#### Publication bias

**Fig. 4:**
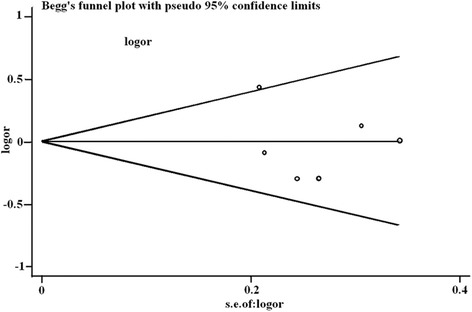
Begg’s funnel plot of the identified studies for the CCL2-2518A/G heterozygous (AA vs AG) model. CCL2, C-C motif chemokine ligand 2

Publication bias was assessed using the Begg’s test (*P* > │*z*│) and Egger’s test (*P* > │*t*│). The result suggested no significant publication bias (Fig. 4 and Table 3).

## Discussion

**Fig. 5:**
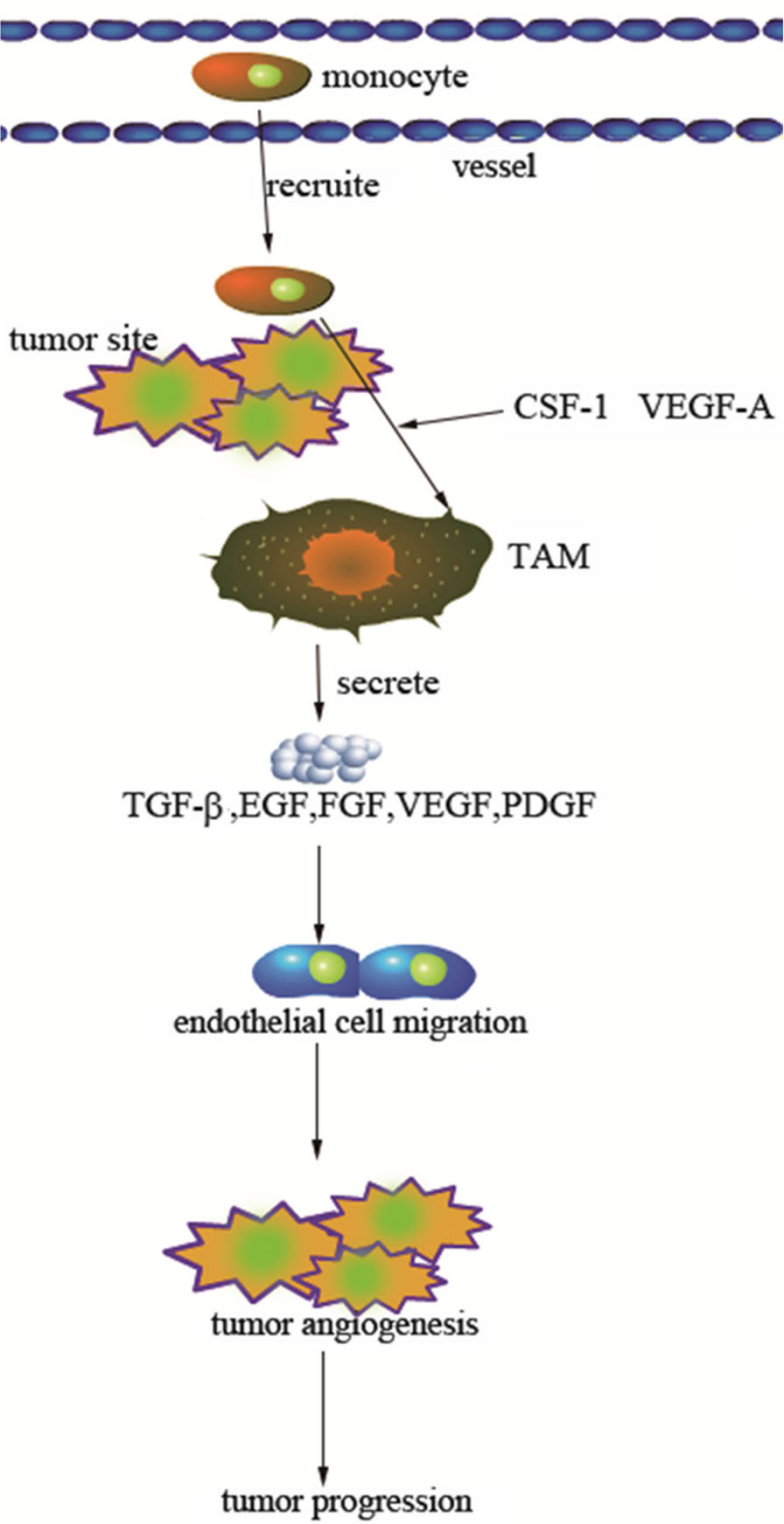
Schematic plot of CCL2 involved in tumor progression. The monocytes expressing CCL2 in blood vessel were recruited to tumor site through CCR2, TAMs when stimulated by cell factors (M-CSF-1, VEGF-A, IL-4, IL-10, IL-13), then TAMs induce endothelial cell migration by secreting TGF-β, EGF, FGF, VEGF, and PDGF, and these TAM-derived factors could promote tumor angiogenesis and CCL2-involved tumor progression. CCL2 chemokine (C-C motif) ligand 2, CCR2 CC chemokine receptor, TAMs tumor-associated macrophages, M-CSF macrophage colony-stimulating factor, VEGF vascular permeability factor, IL interleukin

CCL2 is a member of the C-C chemokine family and involved in the tumorigenesis and metastasis of tumor by participating in mediating the tumor microenvironment [4]. CCL2 mediates the tumor generation and metastasis through multiple mechanisms, such as inducing tumor angiogenesis, mediating tumor immune response, promoting the tumor invasion and metastasis, and directly contributing to tumor progression [21]. The role of CCL2 in the development of tumor is mediated through its receptor CCR2. CCL2 recruits monocytes with CCR2 from the peripheral blood to tumor site and interacts with them. Subsequently, the recruited monocytes transform into TAMs stimulated by cell factors (like macrophage colony-stimulating factor (M-CSF), vascular permeability factor (VEGF)-1, IL-4, IL-10, IL-13), and then, tumor angiogenesis is induced by TAM-secreted angiogenesis factors (Fig. 5) [22, 23]. Of note, CCL2 stimulates tumor cell proliferation and migration by activating IP3-dependent Akt/PKB signal pathway [24]. And CCL2-CCR2 axis also promotes metastasis of tumor cell by activating ERK1/2-MMP2/9 signal pathway [25]. Previous researches have assessed the association between CCL2-2518A/G (rs1024611) polymorphism and cancer risk, including breast cancer, lung cancer, gastric cancer, bladder cancer, ovarian cancer, and others. Sambyal et al. showed that GG genotype of the CCL2-2518A/G polymorphism was a risk factor for breast cancer in Punjab [12]. However, Kruszyna et al. found that the CCL2-2518A/G polymorphism was not associated with the breast cancer risk [17]. Thus, we performed this meta-analysis to assess the association between CCL2-2518A/G and gynecological cancer risk.

This study identified CCL2-2518A/G genetic polymorphism is associated with gynecological cancer risk. The result showed that the homozygote GG, not AA or AG + GG, was a risk factor for gynecological cancer. In subgroup analysis by ethnicity, we found a distinct discrepancy between Asian and European population. Individuals carried AA or A genotype may have a decreased risk for gynecological cancer in Asians but AA genotype is a risk factor in Europeans, and the reason is unclear. This suggests that AA or A genotype may be a protective factor for gynecological cancer in Asians but AA genotype a risk factor in Europeans. In contrary to the finding of this study, Jia et al. conducted a meta-analysis of 11 studies on several types of cancer such as oral cancer, cervical cancer, breast cancer, and bladder cancer and demonstrated that the GG genotype was actually associated with the reduced overall cancer risk in Asian population [26]. However, this report only contained two (2/11) types of gynecological cancer, so we do not think the conclusion was applicable for all gynecological cancer types. These results suggested that the role of GG or G genotype in the incidence of gynecologic cancer in the Asians should be further investigated in future studies with sufficient sample size. Even so, the significant difference of A vs G in Asians indicated that allele A of rs1024611 seems to play a protective role comparing to G allele, and measures targeting this SNP may provide benefits in preventing the occurrence of gynecological cancer.

Although this meta-analysis included five genetic models, there are several limitations. First, because this study was used to analyze the association between CCL2 and gynecological cancer risk, the number of articles identified is relatively smaller than other researches that explored all types of cancers. Second, one article showed genotype frequency in the control group deviated from HWE, which might have affected the results of the original study and this meta-analysis. Third, while there were five studies conducted in Asian countries, there was only one study in Europeans. Thus, the subgroup analysis results may not be reliable.

## Conclusion

The meta-analysis demonstrated a noteworthy association between CCL2-2518A/G polymorphism and the risk of gynecological cancer, and the association varied by ethnicity. In the overall analysis, the allele GG of rs1024611 was associated with an increased risk of gynecological cancer when compared to AA or AG + AA. However, in subgroup analysis, AA or A genotype was associated with a decreased risk of gynecological cancer in Asians compared with AG + GG, GG, and G, while AA was associated with an increased risk when compared with AG in Europeans. To further confirm these associations, studies with large sample size, involving different cancer types and multiple ethnic groups, should be considered.

## Abbreviations

BC: Breast cancer
CCL2: Chemokine (C-C motif) ligand 2
CCR2: CC chemokine receptor
CNKI: Chinese National Knowledge Infrastructure
EC: Endometrial carcinoma
HWE: Hardy-Weinberg equilibrium
IL: Interleukin
LPS: Lipopolysaccharide
MCP-1: Monocyte chemoattractant protein-1
M-CSF: Macrophage colony-stimulating factor
NK: Natural killer
OC: Ovarian cancer
ORs: Odds ratios
PRISMA: Preferred reporting items for systematic reviews and meta-analyses
SNP: Single nucleotide polymorphism
TAMs: Tumor-associated macrophages
UCC: Uterine cervix cancer
VEC: Vascular endothelial cell
VEGF: Vascular permeability factor
